# A cross-sectional study to evaluate second line virological failure and elevated bilirubin as a surrogate for adherence to atazanavir/ritonavir in two urban HIV clinics in Lilongwe, Malawi

**DOI:** 10.1186/s12879-017-2528-0

**Published:** 2017-07-03

**Authors:** Dennis Miyoge Ongubo, Robertino Lim, Hannock Tweya, Christopher Chikhosi Stanley, Petros Tembo, Richard Broadhurst, Salem Gugsa, McNeil Ngongondo, Colin Speight, Tom Heller, Sam Phiri, Mina C. Hosseinipour

**Affiliations:** 1 Tulane University School of Public Health and Tropical Medicine, New Orleans, USA; 2 Tufts University School of Medicine, Boston, USA; 3 Lighthouse Trust, Lilongwe, Malawi; 4 The International Union Against Tuberculosis and Lung Disease, Paris, France; 5 University of North Carolina Project, Lilongwe, Malawi; 6 University of North Carolina School of Medicine, Chapel Hill, USA; 7 Department of Public Health, University of Malawi, College of Medicine, School of Public Health and Family Medicine, Lilongwe, Malawi

**Keywords:** Virological failure, Atazanavir/ritonavir, Second line, Antiretroviral therapy, Bilirubin, Adherence, Surrogate

## Abstract

**Background:**

Malawi’s national antiretroviral therapy program provides atazanavir/ritonavir–based second line regimens which cause concentration-dependent rise in indirect bilirubin. We sought to determine if elevated bilirubin, as a surrogate of atazanavir/ritonavir adherence, can aid in the evaluation of second line virological failure in Malawi.

**Methods:**

We conducted a cross-sectional study of HIV-infected patients ≥15 years who were on boosted protease inhibitor-based second line antiretroviral therapy for at least 6 months in two urban HIV clinics in Lilongwe, Malawi. Antiretroviral therapy history and adherence data were extracted from the electronic medical records and blood was drawn for viral load, complete blood count, total bilirubin, and CD4 cell count at a clinic visit. Factors associated with virological failure were assessed using multivariate logistic regression model.

**Results:**

Out of 376 patients on second line antiretroviral therapy evaluated, 372 (98.9%) were on atazanavir/ritonavir-based therapy and 142 (37.8%) were male. Mean age was 40.9 years (SD ± 10.1), mean duration on second line antiretroviral therapy was 41.9 months (SD ± 27.6) and 256 patients (68.1%) had elevated bilirubin >1.3 mg/dL. Overall, 35 (9.3%) patients had viral load >1000 copies/ml (virological failure). Among the virologically failing vs. non-failing patients, bilirubin was elevated in 34.3% vs. 72.0% respectively (*p* < 0.001), although adherence by pill count was similar (62.9% vs. 60.7%, *p* = 0.804). The odds of virological failure were higher for adults aged 25–40 years (adjusted odds ratio (aOR) 2.5, *p* = 0.048), those with CD4 cell count <100 (aOR 17.5, *p* < 0.001), and those with normal bilirubin levels (aOR 5.4, *p* < 0.001); but were lower for the overweight/obese patients (aOR 0.3, *p* = 0.026). Poor pill count adherence (aOR 0.7, *p* = 0.4) and male gender (aOR 1.2, *p* = 0.698) were not associated with second line virological failure.

**Conclusions:**

Among patients receiving atazanavir/ritonavir-based second line antiretroviral therapy, bilirubin levels better predicted virological failure than pill count adherence. Therefore, strategic use of bilirubin and viral load testing to target adherence counseling and support may be cost-effective in monitoring second line antiretroviral therapy adherence and virological failure. Drug resistance testing targeted for patients with virological failure despite elevated bilirubin levels would facilitate timely switch to third line antiretroviral regimens whenever available.

## Background

Antiretroviral therapy (ART) scale-up in developing countries reversed upward trends in HIV-associated mortality, morbidity, and disease transmission (http://www.unaids.org/sites/default/files/country/documents/MWI_narrative_report_2015.pdf). Despite these improved outcomes in many patients on ART, some eventually fail first line and second line treatment resulting in increased morbidity and mortality for people living with HIV (PLHIV) [1, 2]. Monitoring of treatment adherence and treatment failure is a challenge (http://www.who.int/hiv/pub/arv/adult2010/en/; http://www.who.int/hiv/topics/treatment/First_Line_ART_failure_RLS_metanalysis.pdf [3]), particularly due to the limited access to viral load testing and genotyping assays [4, 5]. Poor adherence to first line ART often translates to continued poor adherence to later lines of ART [6, 7]. While drug resistance contributes to second line virological failure (VF) [8], poor adherence, often in the absence of drug resistance has been described [9–13]. Therefore, detection of poor adherence to ART accompanied by targeted adherence counseling and support may lead to better virological suppression and lower ART switch rates [14, 15].

Approximately 1% of patients in Malawi’s national ART program were on second line regimens with the majority (91%) assessed as adherent based on indirect medication adherence measurements such as pill count and self-reported missed doses (http://www.unaids.org/sites/default/files/country/documents/MWI_narrative_report_2015.pdf). Malawi adopted atazanavir/ritonavir (ATV/r) as the boosted protease inhibitor (PI/r) of choice in 2013; this being a switch from lopinavir/ritonavir (LPV/r) used earlier in its second line ART regimens [16]. ATV/r causes a dose-dependent indirect hyperbilirubinemia [17–21]. This biologic phenomenon has the potential, as an inexpensive surrogate measure of adherence to an ATV/r regimen [22], to distinguish between non-adherence and drug resistance as the cause for VF of second line ART regimens.

There is currently limited data describing both second line VF and whether ATV/r-associated hyperbilirubinemia might serve as a biologic marker of treatment adherence. We, therefore, sought to determine the prevalence and understand the factors associated with VF in a cohort of HIV-infected ART-experienced Malawians on second line ART. We also sought to evaluate the role of serum total bilirubin as a surrogate marker for ATV/r adherence.

## Methods

### Study setting

This study was conducted at Lighthouse (LH) and Martin Preuss Centre (MPC) outpatient HIV clinics in Lilongwe, Malawi. The Lighthouse Trust, operating both LH clinic and MPC, is the largest provider of adult HIV care for PLHIV in Malawi. The integrated Tuberculosis(TB)/HIV care program and Option B+ program for HIV positive pregnant and breastfeeding women and their families is based at MPC, while all other services are provided at both sites. Viral load (VL) monitoring is done according to Malawi’s national ART guidelines at 6 months following initiation of a new ART regimen and at 2 years and every 2 years thereafter [16]. First line ART is tenofovir/lamivudine/efavirenz (TDF/3TC/EFV); referred to within the Malawi National ART Program as 5A) with the potential use of nevirapine (NVP), abacavir/lamivudine (ABC/3TC) or zidovudine/lamivudine (AZT/3TC) for toxicity management ([16], http://www.who.int/hiv/pub/guidelines/arv2013/download/en/). Second line ART is AZT/3TC or TDF/3TC plus ATV/r. Lopinavir/ritonavir (LPV/r) is available as a substitute for ATV/r’s toxicity or when TB treatment is required ([16], http://www.who.int/hiv/pub/guidelines/arv2013/download/en/).

### Study population and design

Study participants were identified from the clinics’ Electronic Medical Records (EMR) systems. They included HIV-infected patients aged ≥15 years attending the MPC and LH outpatient HIV clinics who were receiving PI/r-based second line ART for at least 6 months and with HIV-1 RNA (VL) results. At their regularly scheduled outpatient visits between December 2013 and May 2014, blood was collected for VL, complete blood count (CBC), CD4 cell count, renal function tests and liver panel. We then conducted a cross-sectional study of these participants to assess the factors associated with second line VF within these two facilities. At the time of evaluation, it was estimated that approximately 400 individuals had been initiated on second line therapy in Lighthouse clinics (approximately 2% of the Lighthouse ART population). This proportion is slightly higher than the national average (1% of the national ART population) because Lighthouse, as a center of excellence, serves as a referral centre as well as one of the first and largest providers of ART services in the country.

### Sources of data

All HIV-infected individuals receiving care at LH and MPC were registered in the EMR systems, as described previously ([23], https://github.com/openmrs), where detailed patients’ records collected were accessible through printed reports or directly via the touchscreen terminals in the reception and clinical consultation rooms. These systems also guided the healthcare workers according to Malawi’s clinical HIV guidelines and facilitated medication prescribing and dispensing while performing clinical calculations (e.g., drug adherences). For this study, socio-demographic, clinical and laboratory data, body weight, ART treatment history and previous trends in antiretroviral drug adherence (through pill counts) for individuals on second line ART were obtained from the EMR systems and patients’ medical charts.

### Laboratory evaluations

All the blood samples were processed at the University of North Carolina Research Project Laboratory in Lilongwe. VL was measured using the Abbott Real Ti*m*e HIV-1 assay system (https://www.abbottmolecular.com/us/products/realtime-hiv-1.html). CD4 cell counts were determined by flow cytometry using the Becton Dickinson FACSCount system (Becton Dickinson, Mountain View, California, USA). Complete blood count (CBC) was analyzed in the Beckman Coulter AcT 5diff Cap Pierce hematology analyzer (Beckman Coulter, Miami, FL), while liver and renal function tests were analyzed in the Roche Cobas C 311 chemistry analyzer. Laboratory results were graded according to the National Institute of Health Division of AIDS (DAIDS) Tables for grading the severity of adult and pediatric adverse events: Version 2.0 - November 2014 (http://rsc.tech-res.com/Document/safetyandpharmacovigilance/DAIDS_AE_GRADING_TABLE_v2_NOV2014.pdf).

### Variables

The primary outcome was the proportion of patients on second line PI/r-based ART with virological failure (VF) defined as VL >1000 copies/ml per Malawi’s guidelines [16] based on a single blood draw regardless of reported pill count adherence*.* The primary predictor variable was the cumulative PI/r pill count adherence, whenever possible, which is the average PI/r pill count adherence for at least 6 months prior to the VL sample collection date. This is calculated as:

(pill adherence) = (pill sum difference) / (pill use interval), where;

- ➢Pill sum difference = (previous pill sum) – (current pill count)
- ➢Previous pill sum = (pill count prior to supply visit) + (pills dispensed at supply visit)
- ➢Pill use interval = (no. of days used pills since last supply) X (number of PI/r pills taken per day)

We defined good adherence for desirable virological outcome (VL ≤ 1000 copies/ml) in this study as average pill count adherence of ≥95% and ≤105% [24]. The other variables of interest included age, gender, body mass index (BMI), CD4 cell count, total bilirubin, duration on ART, duration on second line ART, PI/r base, and nucleoside reverse transcriptase inhibitor (NRTI) backbone. All study participants analyzed had VL measurements for determination of VF. We also used a VL threshold of 400 copies/ml to calculate the prevalence rate of VF for comparison to other studies.

### Statistical analysis

Descriptive statistics were used to summarize the study population characteristics and the overall prevalence of second line VF. Continuous variables were summarized using mean and standard deviation (SD); categorical variables were summarized using frequency and percent (%). The distribution of viral load measurement in our study sample was non-normal due to the high proportion of individuals that were successfully suppressed. Other variables were normally distributed. Associations between socio-demographic or HIV-related variables and VF were assessed using the χ^2^ test for categorical variables and the Student’s *t*-test for continuous variables. We used univariate logistic regression analysis to identify factors associated with VF, in addition to the known determinants of VF [25, 26]. Sensitivity, specificity, positive and negative predictive values using bilirubin testing to screen for VF were also calculated. We then used a multivariate logistic regression model fitted through backward selection of variables to assess the adjusted effect of predictor variables on the primary outcome (VF). Age, gender, pill count adherence and time on ART were included a priori to the model. Variance inflation factors were computed for each of the predictor variable to exclude from the model covariates that demonstrated collinearity. All analyses were done in STATA SE version 12.0 (College Station, Texas). Statistical significance was considered at a two-sided α-level of 0.05.

## Results

Out of 376 HIV-infected patients on second line ART evaluated, 372 (98.9%) were on ATV/r-based therapy at the time of study and 142 (37.8%) were male (Table 1). The mean age was 40.9 years (SD ± 10.1), the mean duration on second line ART was 41.9 months (SD ± 27.6), and the mean duration on ATV/r-based second line therapy was 9.1 months (SD ± 1.5). Two hundred and twenty nine (60.9%) patients appeared to have averaged good adherence by pill count over the previous 6 months while 256 (68.1%) patients had elevated bilirubin (>1.3 mg/dL). Of the 376 individuals with VL data, 35 (9.3%) had VL > 1000 copies/ml, 37 (9.8%) had VL ≥ 400 copies/ml while 93 (24.7%) had VL ≥40 copies/ml.

**Table 1:** Population characteristics of HIV-infected patients on second line ART in two urban HIV clinics in Lilongwe, Malawi

Characteristics (Categorical)	N = 376 *n* (%)
Age in years	
15–24	27 (7.2)
25–40	161 (42.8)
> 40	188 (50.0)
Gender	
female	234 (62.2)
male	142 (37.8)
CD4 cell count (cells/mm^3^)	
≥ 100	362 (96.3)
< 100	12 (3.2)
Virological failure (>1000 copies/ml)	
yes	35 (9.3)
no	341 (90.7)
BMI (kg/m^2^)	
normal	207 (55.1)
underweight	25 (6.7)
overweight/obese	144 (38.3)
Pill count adherence	
good (95% - 105%)	229 (60.9)
poor (<95% or >105%)	147 (39.1)
Current boosted protease inhibitor	
atazanavir/ritonavir	372 (98.9)
lopinavir/ritonavir	4 (1.1)
Current NRTI backbone	
ABC/3TC	5 (1.3)
AZT/3TC	67 (17.8)
TDF/3TC	303 (80.6)
d4T/3TC	1 (0.3)
Reason for ART initiation	
WHO clinical stage III/IV	283 (75.3)
CD4 cell count below threshold	74 (19.7)
pregnancy/lactating	3 (0.8)
Total bilirubin level	
elevated (>1.3 mg/dL)	256 (68.1)
normal (≤1.3 mg/dL)	118 (31.4)
Characteristics (Continuous)	*N* = 376
	Mean (SD)
age (years)	40.9 (10.1)
CD4 cell count (cells/mm^3^)	442.6 (245.8)
time on ART (months)	89.9 (34.4)
time on second line ART (months)	41.9 (27.6)
time on ATV/r base (months)	9.1 (1.5)
BMI (kg/m^2^)	24.7 (5.0)
total bilirubin (mg/dL)	2.2 (1.6)
hemoglobin (g/dL)	12.9 (2.1)

Numbers and proportions may not add up due to missing data

Overall, out of 35 (9.3%) patients who had Malawi-defined VF (VL > 1000 copies/ml) while on second line ART (Table 2), the majority (60%) were aged 25–40 years old in these two urban HIV clinics. Among the virologically failing vs. non-failing patients, those failing therapy were younger (mean age of 36 vs. 41.4 years, *p* = 0.0025), had lower CD4 cell count (mean of 206 vs. 466 cells/mm^3^, *p* < 0.001), and had significantly lower bilirubin levels (1.0 vs. 2.4 mg/dL, *p* < 0.001). Bilirubin was elevated in 34.3% vs. 72.0% respectively (*p* < 0.001), although adherence by pill count were similar (62.9% vs. 60.7%, *p* = 0.804). The distributions of other population characteristics were similar among the virologically failing vs. non-failing patients. We observed medium correlation between VF and World Health Organization (WHO) defined immunological failure (CD4 cell count <100 cells/mm^3^) (*r =* 0.3119, *p-*value <0.001).

**Table 2:** Characteristics of HIV-infected patients with second line virological failure in two urban HIV clinics in Lilongwe, Malawi

Characteristics (Categorical)	Viral load ≤ 1000 *N* = 341 *n(column%)*	Viral load > 1000 *N* = 35 *n(column%)*	Total *N* = 376 *n(column%)*	*p*-value
Age in years				
15–24	22 (6.5)	5 (14.3)	27 (7.2)	
25–40	140 (41.1)	21 (60.0)	161 (42.8)	
> 40	179 (52.5)	9 (25.7)	188 (50.0)	0.005*
Gender				
female	212 (62.2)	22 (62.9)	234 (62.2)	
male	129 (37.8)	13 (37.1)	142 (37.8)	0.936
CD4 cell count (cells/mm^3^)				
≥ 100	335 (98.5)	27 (79.4)	362 (96.8)	
< 100	5 (1.5)	7 (20.6)	12 (3.2)	<0.001*
BMI (kg/m^2^)				
normal	183 (53.7)	24 (68.6)	207 (55.1)	
underweight	21 (6.2)	4 (11.4)	25 (6.7)	
overweight/obese	137 (40.1)	7 (20)	144 (38.3)	0.031*
Pill count adherence				
good (95% - 105%)	207 (60.7)	22 (62.9)	229 (60.9)	
poor (<95% or >105%)	134 (39.3)	13 (37.1)	147 (39.1)	0.804
Current boosted protease inhibitor				
atazanavir/ritonavir	338 (99.1)	34 (97.1)	372 (98.9)	
lopinavir/ritonavir	3 (0.9)	1 (2.9)	4 (1.1)	0.325*
Current NRTI backbone				
ABC/3TC	4 (1.2)	1 (2.9)	5 (1.3)	
AZT/3TC	61 (17.9)	6 (17.1)	67 (17.8)	
TDF/3TC	275 (80.7)	28 (80)	303 (80.6)	
d4T/3TC	1 (0.3)	0 (0)	1 (0.3)	0.583*
Reason for ART initiation				
WHO clinical stage III/IV	254 (77.9)	29 (85.3)	283 (78.6)	
CD4 count below threshold	69 (21.2)	5 (14.7)	74 (20.6)	
pregnancy/lactating	3 (0.9)	0 (0)	3 (0.8)	0.633*
Total bilirubin level				
elevated (>1.3 mg/dL)	244 (72.0)	12 (34.3)	256 (68.5)	
normal (≤1.3 mg/dL)	95 (28.0)	23 (65.7)	118 (31.6)	<0.001
Characteristics (Continuous)	Viral Load ≤ 1000	Viral Load > 1000		*p*-value
	*Mean (SD)*	*Mean (SD)*	Total	
age (years)	41.4 (10.0)	36.0 (9.5)	376	0.0025
CD4 cell count (cells/mm^3^)	466.3 (241.2)	205.6 (146.5)	374	<0.001
time on ART (months)	90.1 (34.9)	88.5 (29.4)	372	0.795
time on second line ART (months)	42.5 (27.7)	35.5 (25.9)	376	0.1516
time on ATV/r base (months)	9.1 (1.5)	9.0 (1.8)	372	0.7069
BMI (kg/m^2^)	24.9 (5.1)	23.2 (4.7)	376	0.0516
total bilirubin (mg/dL)	2.4 (1.6)	1.0 (1.1)	374	<0.001
hemoglobin (g/dL)	13 (2.1)	12.1 (1.5)	376	0.0144

*denotes Fisher’s exact test

The sensitivity and specificity of using bilirubin testing at the 1.3 mg/dL level to screen for VF was 65.7% and 72% respectively with a false negative rate of 34.3% and false positive rate of 28%. Bilirubin testing had a negative predictive value (NPV) of 95.3% and a positive predictive value (PPV) of 19.5%. The positive likelihood ratio (PLR) was 2.3 while the negative likelihood ratio (NLR) was 0.5. Using higher bilirubin cutpoints increased sensitivity but reduced specificity (Table 3).

**Table 3:** Association of different bilirubin cut-off levels with second line virological failure

Bilirubin levels*	Sensitivity	Specificity	Positive Predictive Value	Negative Predictive Value
1.3 (Lab Upper Limit of Normal)	65.7	72	19.5	95.3
<2.08 (DAIDS Toxicity Grade < =1)	85.7	49.9	15	97.1
<3.38 (DAIDS Toxicity Grade < =2)	94.3	20.1	10.9	97.1
<6.5 (DAIDS Toxicity Grade < =3)	100	3.24	9.6	100

*Bilirubin values above represent the cut-offs corresponding with DAIDS Version 2.0 toxicity reporting requirements

Univariate and multivariate logistic regressions identified factors associated with VF (Table 4). The odds of VF were higher for adults aged 25–40 years (adjusted Odds Ratio (aOR) 2.5, *p* = 0.048), those with CD4 cell count <100 cells/mm^3^ (aOR 17.5, *p* < 0.001), and those with normal bilirubin levels (aOR 5.4, *p* < 0.001); but were lower for the overweight/obese patients (aOR 0.3, *p* = 0.026). Poor adherence by pill count (aOR 0.7, *p* = 0.4) and male gender (aOR 1.2, *p* = 0.698) were not associated with increased risk of VF. Similarly, time on second line ART (aOR 1; *p* = 0.921) and time on ATV/r (aOR 0.9; *p* = 0.409) were not associated with increased risk of VF and did not demonstrate collinearity. Additionally, using bilirubin as a continuous variable, the odds of VF were 0.31 less for every unit rise in total bilirubin (*p*-value <0.005).

**Table 4:** Factors associated with second line virological failure in two urban HIV clinics in Lilongwe, Malawi

	Un-adjusted		Adjusted	
Characteristics	Odds Ratios	*p*-value	Odds Ratios	*p*-value
Age in years (at time of study evaluation)				
> 40	Referent			
25–40	3	0.008	2.5	0.048
15–24	4.5	0.012	1.5	0.611
age, per each additional year	0.9	0.003		
age at second line ART initiation, per additional year	<1.0	0.008		
Gender				
female	Referent			
male	<1.0	0.936	1.2	0.698
Total time on ART, per additional month	<1.0	0.794		
Time on second line ART, per additional month	<1.0	0.154	1	0.921
Time on ATV/r, per additional month	<1.0	0.706	0.9	0.409
BMI (kg/m^2^)				
normal	Referent			
underweight	1.5	0.525	0.4	0.235
overweight/obese	0.4	0.034	0.3	0.026
Current boosted protease inhibitor				
ATV/r	Referent			
LPV/r	3.3	0.305		
Current NRTI backbone				
TDF/3TC	Referent			
AZT/3TC	<1.0	0.942		
ABC/3TC	2.5	0.429		
d4T/3TC	1			
CD4 cell count (cells/mm^3^)				
≥ 100	Referent			
< 100	17.4	<0.001	17.5	<0.001
Pill count adherence				
good (95% - 105%)	Referent			
poor (<95% or >105%)	0.9	0.804	0.7	0.4
Total bilirubin, per unit rise (mg/dL)	0.3	<0.001		
Total bilirubin level				
elevated (>1.3 mg/dL)	Referent			
normal (≤1.3 mg/dL)	4.9	<0.001	5.4	<0.001

Variables included in the multivariate analysis that are not known predictors of second line ART failure had *p*-values <0.05 in the univariate analysis. Multicollinearity was assessed using variance inflation factors

## Discussion

We found a surprisingly low prevalence rate of VF (9.3%) among patients retained on second line ART at these two urban outpatient HIV clinics in Lilongwe, Malawi. Importantly, we identified several easily measureable factors predicting VF including bilirubin levels, CD4 cell count, BMI and age. Bilirubin testing, as a biologic marker for adherence to ATV/r-based therapy, may be an effective tool in evaluating patients for ART adherence and VF prior to the more expensive and less available genotype resistance testing and third line therapy.

The prevalence rate of second line VF was lower in this study than reported elsewhere [10], although different VL thresholds were used to make the comparison. We used a VL threshold of 1000 copies/ml to define VF to align to Malawi’s guidelines whereas other studies [2, 27] used a lower VL threshold of 400 copies/ml to define failure. Despite the sensitivity of VF rates to VL thresholds for failure definition [27], our study yielded low rates of 9.8% for VL thresholds of 400 copies/ml. ATV/r which is dosed once daily may have fewer side effects than LPV/r, and this may favor better treatment adherence and virological suppression as evidenced in this study [16, 28]. Previous studies that reported higher rates of VF predominantly used LPV/r as the PI/r of choice [10, 29]. However, we do not expect differences in therapeutic responses to LPV/r versus ATV/r [30]. Improved access to second line ART in Malawi ([2], http://www.who.int/hiv/pub/guidelines/arv2013/download/en/) also may potentially explain the improved virological outcomes evidenced in this study.

Objective biologic measures of ART adherence are more reliable in predicting poor adherence and VF than pill count or patient’s self-report, which often over-estimate adherence levels [29, 31–35]. Therefore, our finding that normal bilirubin values in the setting of ATV/r-based therapy better predicted treatment failure than pill count is not surprising and supports the use of bilirubin as a surrogate for ATV/r adherence and response [36]. Both the lower mean bilirubin and the 2.3 times increased risk for virologically failing patients highlights the usefulness of this test in monitoring second line treatment failure. Patients treated with ATV/r have an increase in unconjugated and total bilirubin levels due to the concentration-dependent ATV/r inhibition of the enzyme uridine diphosphate glucuronosyltransferase 1A1 (UGT1A1) responsible for bilirubin conjugation [17–19]. Despite transient fluctuations of serum bilirubin levels over time, the elevation often remains stable for at least two years after initiation of ATV/r [36] making it useful for adherence monitoring in ART-experienced patients [20, 37]. In countries using ATV/r based second line therapy, a cost-effective algorithm incorporating targeted bilirubin and drug resistance testing into routine VL monitoring could be developed to monitor second line VF and promote treatment switch. In that algorithm, patients with poor adherence based on low bilirubin levels are targeted for intensive adherence counseling and support. Among those with elevated bilirubin and hence presumed good adherence, in addition to standard adherence counseling, drug resistance assays would be conducted to detect potential drug resistance. Only those failing treatment due to resistance mutations would switch to third line ART. As resistance testing and third line ART are relatively expensive, such an algorithm would address adherence issues, target those most likely to have resistance, and more promptly identify those with resistance in need of third line treatment. This strategy may potentially yield good virological outcomes [38] and increase survival rates [39, 40] in this target population. As fewer samples for resistance testing would be taken from patients failing second line ART simply due to poor adherence, this would increase the proportion of samples with significant resistance mutations where an effective third line treatment regimen would need to be determined.

Lower CD4 cell count was significantly associated with VF in this study. A previous study conducted within Malawi’s national ART program had indicated an association of lower CD4 cell count (<100cells/ml) with high levels of first line ART resistance, possibly contributing to second line ART failure [41]. However, studies in other resource-limited settings have demonstrated poor positive predictive values of WHO CD4 cell count criteria (<100cells/ml) for virological failure, with the potential for unnecessary ART switch [42–45]. Adults aged 25–40 years old, being majority among those failing therapy in these facilities, independently predicted VF thus supporting findings from other studies [46]. These individuals are more likely to miss clinic appointments and hence less likely to be adherent to ART [47]. Similarly, increasing age may be associated with greater maturity and understanding of health issues, and established lifestyles. These factors may likely influence health-seeking behaviors, long-term adherence to ART and virological suppression. We may not have seen a significant relationship in adolescents aged 15–24 years old due to small sample size of this age group. Body mass index (BMI) frequently used for monitoring clinical progression of HIV/AIDS demonstrated an association with VF. Low BMI has been linked with HIV-associated morbidity and mortality [2], whereas weight gain while on ART may indicate optimal medication adherence. Despite varied views on the use of clinical markers to predict VF [48, 49], high BMI (overweight and obesity) was significantly associated with VL ≤1000, perhaps relating the general health improvements with favorable ART response in this population. However, the large proportion of second line ART patients classified as overweight or obese in this study may pose future health concern due to non-communicable diseases. Gender was not independently associated with VF in spite of the perceived vulnerabilities of men to ART failure [50–52]. Similarly, the duration of second-line ART was not associated with VF [2], probably reflecting the high genetic barrier to viral resistance accorded by protease inhibitors in the context of good adherence [53].

The findings of this study should be considered in light of their strengths and limitations. Firstly, there is the likelihood of selection bias. Only participants receiving second line ART and who presented to the clinic during the period of the study were included for analysis. Those missing visits, and hence at higher risk for VF due to poor adherence to clinic appointments and missed doses, were not included for the analysis. Therefore, our results represent VF rates among persons on second line ART retained in care and may underestimate the true rate of treatment failure. Secondly, this analysis was based on cross-sectional observational data thereby limiting our ability to determine a causal relationship between laboratory parameters used to monitor second-line ART adherence and VF. Similarly, baseline values which would have improved our understanding of the factors associated with VF by looking at the temporal associations of clinical and laboratory parameters with second line ART, were unavailable. However, because LH and MPC outpatient HIV clinics are major public providers of second line ART in Malawi’s capital city of Lilongwe, the findings in this study could be generalizable for ART programs in Malawi and other regions with a generalized HIV epidemic in urban populations. Thirdly, pill count adherence may be misleading particularly in treatment-experienced patients in whom social desirability bias may encourage pill dumping and lead to overestimation of medication adherence (>105%). Pill count adherence was overestimated in 73 (49.7%) of those with poor pill count adherence estimates. However, just like sub-optimal pill count adherence (<95%), overestimation of adherence has been associated with increased risk of VF [54] thus justifying their inclusion as poor pill count adherence measurements in this analysis. Fourthly, many of the participants were highly experienced on second line ART (42 months) but had much shorter exposure to ATV/r (9 months) due to Malawi’s recent adoption of ATV/r in second line ART regimens. Therefore, we are unable to comment on whether our results would be similar for those with longer duration of ATV/r exposure and if there were transient fluctuations of serum bilirubin over time [36]. Lastly, genotypic resistance testing that is important when selecting alternative therapeutic regimens [55] was not available to characterize the resistance profiles of those with VF, although development of resistance to PIs is uncommon [9]. Resistance testing would have allowed more thorough evaluation of the associations between virological failure, ATV/r adherence and bilirubin levels.

## Conclusions

Given the limited access to VL monitoring, drug resistance testing and third line treatment regimens in sub-Saharan Africa, strategies for cost-effective delivery of ART services are needed. Bilirubin, as a surrogate marker for ATV/r adherence among patients receiving second line therapy, better predicted VF than pill count adherence. Strategic use of bilirubin and VL testing to target adherence counseling and support may be cost-effective in monitoring second line ART adherence and VF particularly for adults aged 25–40 years and those with CD4 cell count <100 cells/mm^3^. Therefore, individuals with normal or low bilirubin levels and VF (VL >1000 copies/ml) while on ATV/r-based second line ART would benefit from intensive adherence counseling and support to help optimize the duration of second line treatment regimens. Drug resistance testing targeted for patients with virological failure despite persistently elevated bilirubin levels would facilitate timely switch to third line antiretroviral regimens whenever available.

## Abbreviations

ABC/3TC: Abacavir/Lamivudine
aOR: adjusted Odds Ratio
ART: Antiretroviral therapy
ATV/r: Atazanavir/ritonavir
AZT/3TC: Zidovudine/Lamivudine
BMI: Body Mass Index
CBC: Complete Blood Count
EMR: Electronic Medical Record
LH: Lighthouse
LPV/r: Lopinavir/ritonavir
MPC: Martin Preuss Center
NLR: Negative Likelihood Ratio
NPV: Negative Predictive Value
NRTI: Nucleoside reverse transcriptase inhibitor
NVP: Nevirapine
PI/r: Boosted Protease Inhibitor
PLHIV: People Living with HIV
PLR: Positive Likelihood Ratio
PPV: Positive Predictive Value
TB: Tuberculosis
TDF/3TC/EFV: Tenofovir/Lamivudine/Efavirenz
VF: Virological Failure
VL: Viral load
